# Progress in Medicine

**Published:** 1898-12-31

**Authors:** 


### PROGRESS IN MEDICINE.

### DISEASES OF THE BLOOD.

(*Continued from page* 131.)

**Treatment of Anæmia.**—The effect of high altitudes on anæmia is discussed by Lepine.[17] Muntz found that the blood of animals on mountains contains much more oxygen, iron, and fixed matter than does that of the same animals on plains. There is a similar increase when the animals are kept in a chamber at a low pressure. In either case the red corpuscles increase more quickly than the hæmoglobin, and numbers of small and nucleated red corpuscles appear, which are probably immature growing ones, but there is later on a great increase of hæmoglobin. This is not due to mere concentration of the blood, for the quantity per kilo of the body weight is greater. In human beings the improvement is retained for some time after a return to the plains, and thus a stay at high altitudes may have great value in arresting anæmia by affording a temporary stimulus to the blood-forming organs. The writer confesses that the means by which the effect is produced is unknown, though the result is well proved.

In the treatment of chlorosis Syers[18] remarks that apparently anæmic persons have sometimes normal amounts of hæmoglobin. Hence we ought to test such blood, to avoid uselessly treating these persons with iron. Again, the hæmoglobin sometimes increases very fast without a change in the pallor of the complexion; but if treatment be stopped too soon, these patients at once fall back. He insists on the need of removing dyspepsia before beginning iron in chlorosis, and prefers the sulphate of iron with sulphuric acid and a few drops of the liquor arsenici hydrochloricus; or else fresh reduced iron. It is useful to estimate hæmoglobin from time to time, and to examine the corpuscles for poikilocytosis, to exclude pernicious anæmia, though counting is not usually necessary. Warfringe[19] speaks in favour of hypodermic administration of iron; and De Dominicis, holding chlorosis to be a specific auto-intoxication, advocates transfusion of blood, which, he finds, affords a lasting cure. W. Edgecombe[20] shows the value of rest in the treatment of chlorosis in lessening the normal destruction of hæmoglobin which occurs daily from exercise. In healthy persons there is a fall in the day and rise in the night of hæmoglobin, the difference being increased if active exercise is taken, while rest reduces the day's fall. Exercise, indeed, in health, produces a great destruction followed by increased production of hæmoglobin, but in chlorosis it is probable that the compensatory reproduction fails. Hence exercise must be curtailed.

In rare cases of pernicious anæmia, enormous doses of iron effect a temporary cure, as in one shown by Sir T. Grainger Stewart.[21] Five times in nine years the patient has come into the Royal Infirmary, Edinburgh, with severe symptoms, and each time has gone out well. Twenty minims of the perchloride were given him every two hours in one of these attacks, when arsenic, bone marrow, and other remedies had failed.

**Nerve Changes.**—An instance of chlorosis in which bilateral foci of red softening occurred in the brain, was seen lately in the London Hospital under the care of Stephen Mackenzie.[22] After two days' malaise the patient became unconscious, the lower extremities were paralysed and flaccid, athetoid movements with some rigidity appeared in the arms, the eyes were turned to the left, and the pupils were unequal. The coma increased, and the patient gradually sank. After death red softening was found on both sides of the brain, involving the internal capsule. J. S. Risien Russell[23] discusses minutely the relation of certain lesions (combined sclerosis) of the spinal cord to anæmia. He holds that the lesions found in this class of cases correspond with those in ataxic paraplegia, except in the far more rapid course of their symptoms. In one of his patients the anæmia was not of the pernicious type, in another the spinal symptoms preceded the anæmia, and in a third death took place before any marked anæmia occurred. He does not, therefore, believe that anæmia is the cause of the spinal changes; nor even that the hæmorrhages produced in various anæmias are the cause of them, for either may occur without the other; nor are degenerations in the vessel walls, or any form of inflammation the cause. Some toxin, in fact, acts on those parts of the cord most easily affected by it. If anæmia is present this lowers the resistance of the nerves, or the same poison may produce anæmia as one of its results. We may here notice a remarkable case of anæmia with purpura and internal hæmorrhages, in which facial paralysis with deflection of the tongue was probably caused by hæmorrhage on the brain. The case is recorded by E. J. Blackell,[24] who says that paralysis and other symptoms gradually disappeared in a month's time.

**The Nature of Leucocytosis.**—At Edinburgh, R. Muir[25] laid down that in this state there is often an increase of the total cells in the body, not merely a local one, amounting at times to double the normal number in a few hours. The increase is almost entirely in the polynuclear cells, and when pus is discharged daily the quantity of these cells formed must be immense. He thinks these cells are formed in the marrow, and enter the blood in their mature state. During suppuration he finds in the marrow great numbers of the finely granular leucoblasts, from which the polynuclear cells are derived. He does not think that there is proof that hyaline cells are also changed into polynuclear. The opposite state, or fewness of leucocytes, may be due to their accumulation in certain localities, to negative chemotaxic substances, and to lesions affecting the marrow and their production there. An increase of the eosinophiles occurs in some malignant diseases, and in affections of the skin and mucous membranes, but their source is uncertain, whether from marrow cells or otherwise.

**Acromegaly.**—Since the great exhibition of cases at the Pathological Society, in November last, several papers have appeared. Furnivall then stated that in all the 34 cases he had analysed there was some affection of the pituitary body, and in many one also of the thyroid. Sternberg divides cases into (1) benign, which may last 50 years; (2) chronic, from 8 to 30 years; and (3) the malignant type, fatal in three or four years. Besides the pituitary theory of its causation, we have the atavistic and the thymus theories, and that of general tissue hypertrophy. Pituitary disease may not be accompanied with typical acromegaly, though, possibly, some of the minor symptoms may be found.

Woods Hutchinson[26] finds that about 250 cases in all have been reported, and notices as the chief symptoms the great and invariable enlargement in length and breadth of the lower jaw, a change which is seen also in the hands, where the growth is less though still marked. Some increase occurs in the feet or the great toe, and in the upper jaw. The superciliary ridges may enlarge and the dorsal spine may be kyphotic. The nose, lips, tongue, palate, uvula, and tonsils may share in the growth. The clavicle, sternum, and ribs (but not the pelvis or the length of the femur) are also often affected. The thymus survives in the adult in some of these patients. They complain of a gradual failure of strength, mental and bodily; the weight is increased, and shooting pains in the vertex, back, and limbs are observed. Diabetes, sweating, Cheynes Stokes' respiration, with coma and collapse, may terminate the scene. Vision may gradually fail, the skin becomes a dirty white colour, thickened and wrinkled, and the hair may be thin and coarse, while the sexual functions gradually cease. Pearce Bailey[27] records a case with enlargement of the thyroid and pituitary bodies. On the latter was an adenomatous outgrowth. He also mentions a patient in whom the enlarged pituitary body contained a hæmorrhage from vascular disease, though no symptoms of acromegaly had occurred, and he thinks this might be an early stage of the disease. In a case at Wandsworth Infirmary, which had lasted about fifteen years, Shattock[18] noticed enlargement and degeneration of the thyroid. The pituitary body consisted of large polyhedral cells with scarcely any stroma. A slight improvement had followed the use of thyroid extract. William Hunter[29] found the P. body highly vascular, and the seat of recent hæmorrhage, the medulla of all the bones examined was hyperæmic, and the thyroid enlarged. Witmer records little or no improvement from the use of thyroid or pituitary extract in a case under his care. H. B. Rolleston[30] found that the headache in two cases was relieved by taking 10 grains daily each of thyroid and pituitary extract, though the skeletal changes were unaltered. Pituitary extract alone has generally failed, while thyroid by itself has given uncertain results to different observers. M. A. Strumpell[31] reports a case with sarcomatous changes in the pituitary body and diabetes, though without any pancreatic lesion, such as has been postulated by Pineles. He considers that in scleroderma we have a disease the direct opposite of acromegaly, a reduction of all those organs which are hypertrophied in the latter. The contrast is like that between myxœdema and Graves' disease. Chadbourne has collected some 14 cases of diabetes in this affection.[32] W. Hutchinson[33] thinks that the skeletal changes occur just at the latest points of development, *i.e.*, at the tips of the segmental crescents of which the body is made up, and supports this view by certain theories of the body arches. He also insists on the close relation which exists between gigantism and acromegaly.

[17] Brit. Med. Jour., June 11. [18] Treatment, Mar. 10. [19] Brit. Med. Jour., June 1. [20] Brit. Med. Jour., June 25. [21] Clin. Jour., Sept. 14. [22] Practitioner, Feb. [23] Lancet, July 2. [24] Lancet, April 16. [25] Brit. Med. Jour., Sept. 16. [26] New York Med. Jour., Mar. 12. [27] Med. Rec., April 16. [28] Lancet, July 23. [29] Brit. Med. Jour., Mar. 19. [30] Lancet, Dec. 4. [31] La Semaine Med., Feb. 26. [32] New York Med. Jour., April 2. [33] Ibid.

### DIPHTHERIA.

(*Continued from page* 213.)

**Serum exanthemata.**—So much confusion exists as to the origin and import of serum exanthemata that the recent articles of Berg will help to clear up many difficulties. Berg[31] cites the report of Hartung[32] showing that in 2,661 injections collected, 294, or 11·4 per cent., developed rashes. In Berg's own hospital, the Willard Parker, in 337 injections, 82 cases of rash were observed, *i.e.*, 24 per cent. of his injections. But

Monti,[33] of Vienna, found the eruption to occur in 52 per cent. of the series of his cases, while Hager[34] did not have a single case of serum exanthema in a series of 61 cases. The variations in the frequency of these rashes in the experiences of different observers, and in that of the same observers following various serums of the same manufacture, seems to indicate that they depend on causes inherent in the serum itself, or on some circumstances connected with the manufacture of the serum. Now Bertin,[35] Sevestre,[36] Johannesen,[37] and others have injected non-immunised horse serum into persons, the two first into patients suffering from diphtheria, the latter into healthy persons, and in all three series erythema was observed, showing that pure horse blood serum does produce eruptions in a large percentage of cases, and that the autoxin present in antitoxic serum does not add to the frequency of their occurrence. But filtering the pure horse serum through a Chamberland filter greatly diminishes the frequency of the eruptions on injection. Berg found that if filtered (with Chamberland filter) antitoxic serum was used in diphtheria cases, the rash only developed in about one-third as many cases as when the serum was unfiltered, or only passed through a coarse filter. In the discussion following Berg's paper, W. H. Park stated that they found in the laboratory there were great differences in the serums taken from different horses as regards the production of rashes, and that in this respect the same horse gave different results at various times. He cited the case of one horse whose serum gave many fewer rashes after it had been sent to the country to recuperate, whereas before, so frequent were the rashes induced that they had to stop using the serum prepared for it. In a general way the larger the dose the more frequent the development of the rashes, and the greater their severity. He did not think that filtering even through a fine filter had been shown to make any difference. About 800 to 1,000 units to the cubic centimetre was as high as it was practicable to get horse serum, though occasionally it might be run up to 1,200 or even 1,500 units.

Northrup also agreed that the size of the dose had much to do with the frequency of the erythemata. Berg classifies the various eruptions thus: (1) Resembling a simple erythema; (2) resembling erythema scarlatiniforme—(*a*) without desquamation, and (*b*) with desquamation; (3) Resembling erythema morbiliforme—(*a*) without desquamation, and (*b*) with desquamation; (4) resembling for the most part erythema multiforme, and including the urticarial cases. (Groups 1 and 4 included the greater number of the cases.) Sometimes the rash comes and goes without altering the course of the case in any way. In the majority a sharp rise of temperature precedes fushers in the occurrence of the rash, and as a rule the temperature declines rapidly with its appearance.

Those cases in which a marked scarlatinal form or measles eruption develops are especially apt to be feverish preceding and during the progress of the eruption, while its disappearance is not marked by a subsidence of the fever. The latter continues and frequently is found to be due to complications of an inflammatory character, involving organs which previous to the appearance of the rash were free from disease. Thus Berg has seen a nephritis, bronchopneumonia, otitis media, and other complications show themselves during the continuance or immediately after the subsidence of the rash. Especially is this true of the polyarthritis so frequently complicating these rashes. These joint pains and inflammations often precede the appearance of the rash and remain during its development and after its subsidence. The appearance of a morbilliform rash has been a factor in cases in which the prognosis was bad.

The rashes may be general, or, as is most frequently the case, localised; in the latter case they are apt to occur round the site of the injection. Localised rashes rarely produce any constitutional disturbance. The rare morbilliform eruption occurs generally in from nine to fourteen days after injection, which Berg remarks corresponds curiously enough to the incubation period of true measles. It may occur as early as the fifth day. When the rash occurs within three to five days it is generally of the scarlatiniform or erythema multiforme variety, the very early rashes being usually the simple form.

Berg gives in tabular form the points on which he is accustomed to rely in differential diagnosis between these rashes and true measles and scarlatina:—

| ANTITOXIN RASH (MORBILLIFORM). | TRUE MEASLES. |
|---|---|
| Prodromal catarrh of conjunctival, nasal, pharyngeal, buccal, and bronchial mucous membranes generally absent. Rash does not appear on face and chest first, but may begin anywhere. | Prodromal catarrhal inflammation. Deep red papular eruption on mucous membranes of soft and hard palate and inside of cheeks. |
| Rash is not uniformly morbilliform over the affected area of skin, but is apt to show a variety of patches of erythema multiforme. | Rash appears on face, neck, and chest first. Uniformly morbilliform, although varying in degree over various portions of body. |
| Eruption disappears after one or more days, and may return after having disappeared entirely. | Duration of rash four or five days, then gradual disappearance. |
| Desquamation not the rule. When it does occur, apt to be coarser than after true measles. | Characteristic desquamation always follows. |

| ANTITOXIN RASH (SCARLATINIFORM). | TRUE SCARLATINA. |
|---|---|
| Vomiting rarely preceding the rash. More or less partial distribution of the eruption. The presence of a non-scarlatiniform eruption, erythema multiforme in character, at isolated areas of skin; frequent disappearance and reappearance of the rash; joint pains early. | Vomiting preceding rash. Eruption much more even, of a uniform character, although varying in degree; duration more than three days; no joint pains at beginning. Does not disappear and reappear at irregular intervals; joint pains late, if any. |
| Duration generally shorter than true scarlatinal rash. | |
| Desquamation rare, and when it occurs much finer than in true scarlatina. | Desquamation in strips. |

A. Jacobi, in the course of the discussion, states that there were two principal forms of rash observed, viz. (1) erythema, and (2) urticaria.

Morse[38] reports a mild case of diphtheria treated with 500 units of serum, which developed urticaria on the seventh day, with glandular swelling, the glands in the groins being as large as walnuts. The thighs became deep purple and the feet enormously swollen. There was almost complete anuria, but no albumen. Recovery in a week.

[31] New York Med. Rec., June. 1898. [32] Die Serum Exanth. bei Diph. Jahr. f. Kindh., Vol. xlii., p. 75. [33] Beitr. zur Anwendung des Heilserumgegen Diph., S. 19. [34] Cent. f. Lun. Med., 1894, No. 48. [35] Gaz Med. de Nantes, 1895. [36] Sem. Med., 1895, No. 17, p. 142. [37] Deut. Med. Woch., 1895, Dec. 19. [33] Bost. Med. and Surg. Jour., Feb. 17, 1898.

